# Ganzfeld Stimulation or Sleep Enhance Long Term Motor Memory Consolidation Compared to Normal Viewing in Saccadic Adaptation Paradigm

**DOI:** 10.1371/journal.pone.0123831

**Published:** 2015-04-13

**Authors:** Caroline Voges, Christoph Helmchen, Wolfgang Heide, Andreas Sprenger

**Affiliations:** 1 Department of Neurology, University Luebeck, Luebeck, Germany; 2 Department of Neurology, General Hospital Celle, Celle, Germany; 3 Institute of Psychology II, University Luebeck, Luebeck, Germany; UMR8194, FRANCE

## Abstract

Adaptation of saccade amplitude in response to intra-saccadic target displacement is a type of implicit motor learning which is required to compensate for physiological changes in saccade performance. Once established trials without intra-saccadic target displacement lead to de-adaptation or extinction, which has been attributed either to extra-retinal mechanisms of spatial constancy or to the influence of the stable visual surroundings. Therefore we investigated whether visual deprivation (“Ganzfeld”-stimulation or sleep) can partially maintain this motor learning compared to free viewing of the natural surroundings. Thirty-five healthy volunteers performed two adaptation blocks of 100 inward adaptation trials – interspersed by an extinction block – which were followed by a two-hour break with or without visual deprivation (VD). Using additional adaptation and extinction blocks short and long (4 weeks) term memory of this implicit motor learning were tested. In the short term, motor memory tested immediately after free viewing was superior to adaptation performance after VD. In the long run, however, effects were opposite: motor memory and relearning of adaptation was superior in the VD conditions. This could imply independent mechanisms that underlie the short-term ability of retrieving learned saccadic gain and its long term consolidation. We suggest that subjects mainly rely on visual cues (i.e., retinal error) in the free viewing condition which makes them prone to changes of the visual stimulus in the extinction block. This indicates the role of a stable visual array for resetting adapted saccade amplitudes. In contrast, visual deprivation (GS and sleep), might train subjects to rely on extra-retinal cues, e.g., efference copy or prediction to remap their internal representations of saccade targets, thus leading to better consolidation of saccadic adaptation.

## Introduction

Saccadic adaptation is a form of implicit motor learning [1] which is required to compensate for physiological changes in saccade performance due to age and growth [2] as well as for consequences of injury [3]. Experimentally, reactive saccades can be adapted using the double-step paradigm introduced by McLaughlin by moving the target during saccade execution outward or inward which is not perceived by the participant due to saccadic suppression [4]. De-adaptation or extinction can be induced by execution of saccades of the same type and vector but without intra-saccadic target displacement (extinction trials) [5,6]. The most important measure for saccade accuracy is the relation between saccade amplitude and target step amplitude, usually referred to as “gain”.

In healthy participants, saccadic adaptation does not lead to an impairment in spatial orientation. Although the motor program for saccade execution has been modified, space is still perceived as stable despite saccade-induced retinal image shifts (spatial constancy). To maintain spatial constancy despite saccade-induce shifts of the retinal image, spatial representation of gaze target is updated (“remapped”). The posterior parietal cortex (PPC) plays an essential role for this remapping [7–9]. However, during saccadic adaptation process, neurons in the lateral intra-parietal sulcus (LIP) [10] still encode the location of the visual saccade target, but not the adapted motor vector of the impending saccade. In addition the cerebellar structures play an important role in saccadic adaptation [11–14] and its consolidation[15]. While PPC is more involved in the control of voluntary saccades, the cerebellum plays important roles in saccadic adaptation [15,16].

Saccade adaptation can be stored. The course of adaptation is a good example of fast motor learning which takes place on the synaptic level [17]. Subsequent testing without intra-saccadic target shift should exhibit similar results as at baseline—if there were no memories of this adaptation process. However, some residual adapted gain is preserved from the end of an adaptation session, referred to as “retention” [18]. Moreover, relearning of the same saccadic adaptation reconsolidates a previous memory trace: adaptation occurs faster which is referred to as “savings” [19–21]. Thus, different learning and memory processes can be obtained from saccadic adaptation over time.

There is an ongoing debate on why modification of saccadic gain by a single course of adaptation—so-called short term saccadic adaptation (STSA) [1]—can still be measured by reflexive saccade paradigms after weeks [22] or even months [23] as long as the same saccade type, vector and amplitude is tested in the same context. This long term saccadic adaptation (LTSA) [20] is a slow-learning process which becomes prominent if retrieval takes place two or more hours later which has been shown similarly for the hand motor domain [24]. A phase of rest or sleep following a learning phase helps to consolidate an acquired skill even by a short period of time [25]. Consolidated skills are usually robust against interference [26]. Models of LTSA has been defined for saccadic [21,27] and other motor adaptation [19,28,29].

Under natural viewing conditions visual cues in our surroundings with distinct stimulus properties and spatial relations among each other serve as contextual cues [13,23]. Consequently eye movements in everyday life are likely to recalibrate the adapted saccadic amplitude. But this does not seem to be the case: there is no reset of the adapted saccadic gain but rather a decrease of adaptation over time [13,22,23]. To elucidate the role of visual stimulation between adaptation learning sessions different forms of visual deprivation have been studied in animal and human studies: When monkeys were visually deprived by opaque goggles or kept in darkness some residual amount of adaptation has been found since the first saccades were not at the gain level reached at the beginning of the previous adaptation session. In awake humans STSA was observed in most of the subjects after a period of visual deprivation (15 min): eye closure or self-generated saccades did not affect the adapted gain in an outward adaptation task [5].

However, long term visual deprivation effects on STSA or LTSA in humans have not been studied so far. Exclusion of visual targets during wakefulness can be achieved by several methods. Keeping the subject in darkness or using opaque goggles prevents visual stimulation but induces fatigue, i.e. a sleep setting. Sleep is a unique condition in which subjects also execute saccades without physically seeing visual stimuli [30]. These rapid eye movements during sleep (REMs) lack of visual feedback and a stable visual surrounding for spatial reference. Hence, sleep can be regarded as a variant of visual deprivation. Recent studies provide evidence that there is neural activity related to visual processing during sleep, especially during dreams (see [31] for review). Furthermore sleep improves memory consolidation in motor and oculo-motor tasks [32,33]. Apart from eye closure visual deprivation can be induced by Ganzfeld stimulation (GS), i.e. a completely homogeneous visual field surrounding the participant induced by white goggles [34].

In this study we therefore investigated the role of natural visual input on the consolidation of adapted saccadic gain. We wanted to know (1) whether free saccadic exploration of a stable natural visual surround—i.e. a constant egocentric visual space—re-adapts the saccadic system and (2) whether visual deprivation or sleep following saccade adaptation facilitates its consolidation. Therefore, we analysed learning curves by the sequence adaptation—extinction—adaptation before and after two hours spent in different visual conditions following adaptation. Measures of savings, i.e. facilitation of saccadic re-adaptation, were of particular interest. Deterioration of re-adaptation has not been described before and will be referred to as ‘less memory savings’. Normal unrestricted viewing served as control condition for two visual deprivation paradigms (sleep and GS). We deprived subjects selectively from visuo-spatial orientation by Ganzfeld stimulation which provides a homogenous illuminative stimulation of the whole visual field, without any fixation cues. We examined long term effects by recording subjects four weeks later again.

## Methods

### Subjects

Thirty-five volunteers without any known neurological or psychiatric disease participated in a saccadic adaptation task with two blocks of experiments and a break in between. They were randomly assigned to 3 groups, differing in how the break between the saccadic adaptation trials was spent: In condition #1 participants were visually deprived using goggles with homogenous Ganzfeld stimulation (GS) during the break of two hours (N = 11, 4 males, 7 females; range 21–25 years; mean age 23 years) whereas in condition #2, 9 other participants (5 males, 4 females; range 19–30 years; mean age 23 years) were allowed to view unrestrictedly during the two hours’ break. In condition #3, fifteen volunteers (6 males, 9 females; range 19–27 years; mean age 23 years) were allowed to sleep. All subjects were students of the University of Luebeck who were naïve about the study's purpose. Their vision was normal or corrected-to-normal at the time of the experiment. Participants' were not allowed to use alcohol or caffeine prior to the experiments. All participants gave written informed consent before starting the experiments corresponding to the declaration of Helsinki. The studies were approved by the Ethics Committee of the University of Luebeck.

### Experimental protocols

Subjects performed the experimental sequence “adaptation—extinction—adaptation” twice, before and after the intervention, i.e. (i) two hours spent in one of the 2 different visual conditions “Ganzfeld stimulation” or “viewing”, or (ii) before and after a whole night of sleep. Saccadic adaptation performance after the intervention was taken as a sign of motor memory consolidation modulated by the visual conditions.

#### Ganzfeld stimulation/viewing condition

Experiments started between 10 a.m. and 1 p.m. Participants conducted two blocks of the experiment (see Table 1) with a two hours’ break in between. The subjects were either visually deprived or allowed to look around. These 2 hours were spent in a quiet, normally illuminated room while listening to calm audio books. Visual deprivation was achieved by goggles with white coloured glasses which induced homogenous Ganzfeld stimulation (GS) [34] without any contours that might enable the subjects to focus. Goggles were put on immediately at the end of the first block and were put off directly before the second part of the oculomotor experiment. Consequently visual input was reduced to a white shade without any edges that may recalibrate the saccadic positioning system. Subjects were asked to keep their eyes open during GS. Subjects in the viewing condition were not allowed to read or to pursue any other activities apart from normal viewing during the two hours’ break. In a previous study we showed that eye movements during mental imagination of semantic contents induce visual scanning behaviour that is comparable to free viewing [30]. In order to induce a “normal” rest situation we did not record eye movements during the two hours break.

**Table 1:** Study design .

**Condition A**	Baseline	Adaptation 1	Extinction	Adaptation 2	**2 h visual deprivation**	Post-adaptation 1	Post-extinction	Post-adaptation 2
**Condition B**	Baseline	Adaptation 1	Extinction	Adaptation 2	**2 h viewing**	Post-adaptation 1	Post-extinction	Post-adaptation 2
**Condition C**	Baseline	Adaptation 1	Extinction	Adaptation 2	**Sleep**	Post-adaptation 1	Post-extinction	Post-adaptation 2

#### Long-term effects

After four weeks all participants of the daytime experiment were recorded once again with the same procedure, but switched visual conditions and adaptation side (cross-over design).

#### Sleep condition

Subjects spent two consecutive nights in our sleep laboratory, the first one for habituation without and the second one (experiment night) with the saccadic adaptation block (“adaptation1—extinction—adaptation2”) in the evening and in the following morning. In both nights, brain, muscle and ocular activity were measured by polysomnography.

In the evening of the experiment night, participants got prepared for the polysomnographic recordings and subsequently performed the first part of the oculomotor experiment. At the end of the block of trials while sitting in the completely dark room of the laboratory, subjects were blindfolded and guided from the oculomotor lab to the sleeping laboratory in the room next door where they laid down to sleep. In the completely darkened sleep laboratory subjects had no visual cues during the night. In the morning while lying in bed again subjects were restricted from viewing and were guided blindfolded to the experiment in the oculomotor lab.

### Experimental setup

In the saccade experiments, subjects sat at a distance of 1.40 m in front of the screen in an otherwise dark room. Head position was stabilized by a chin rest. Subjects were instructed to follow the laser point as quickly and accurately as possible. Saccade stimuli were generated by a red laser point (diameter, 0.1°; laser diode HL 11, LISA Lasersystem, Katlenburg-Lindau; laser intensity 9% of maximum output). The stimuli were back-projected on a translucent screen (Marata screen, BKE, Nörten-Hardenberg, Germany) and controlled by two galvo scanners (GSI Lumonics, Munich, Germany). Eye movements were recorded by a video-based eyetracking system (Eyelink-II, SR Research Ltd., Mississauga, Ontario, Canada) at 500 Hz sampling rate.

In the sleep condition, electroencephalography (EEG), electromyography (EMG) and electrooculography (EOG) were recorded (polysomnography) using Ag/AgCl electrodes. EEG electrodes were placed at positions C3 and C4 of the international 10–20 system. The reference electrode was fixed on the nose. EMG was derived from two electrodes placed on the chin over the right and left mentalis muscle. EOG electrodes were fixed at classical supraorbital and infraorbital positions (for vertical eye movements) of one eye and in the outer angles of both eyes (for horizontal eye movements). A ground electrode was fixed on the forehead. Data were amplified (BrainAmp MRplus, Brain Products, Gilching, Germany) and recorded with a sampling rate of 500 Hz. EOG was DC-recorded, for EEG and EMG, a 250 Hz low-pass filter and a 0.015 Hz high-pass filter were set (time constant 10 s). Sleep was scored offline according to standard criteria [35].

### Experimental paradigms

**Fig 1:**
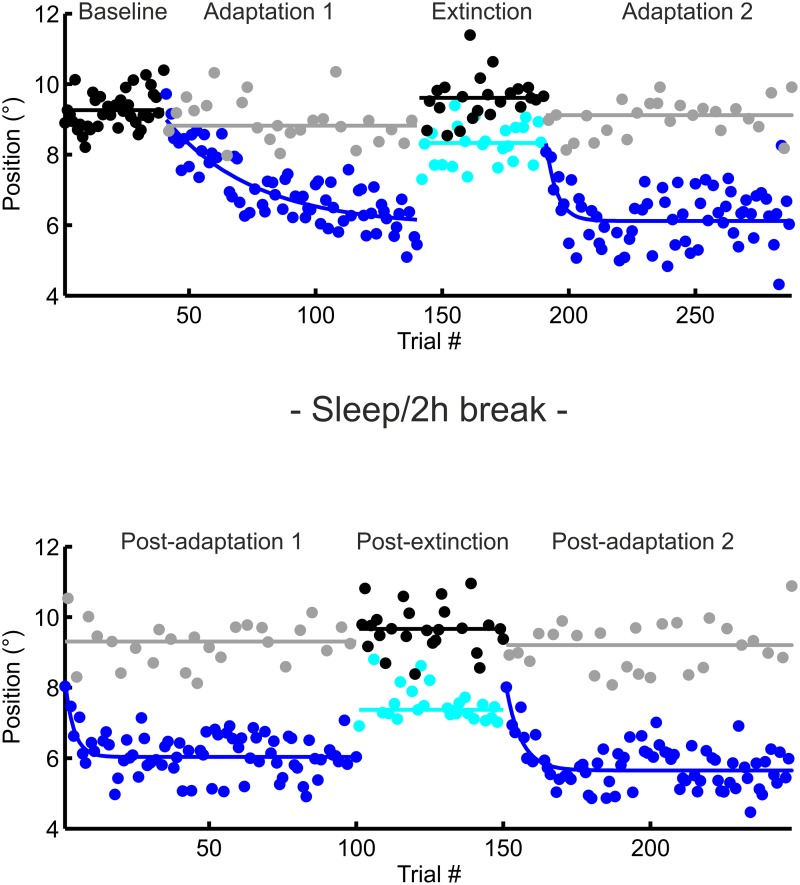
Example data from subject #4, blindfolded condition. Eye position is plotted against trial number, dots symbolise saccadic amplitude. Linear fit was calculated for amplitudes of the non-adapted side (black and gray curves) and exponential fit for the adapted side (light and dark blue curves). Upper part shows data before 2 h of visual deprivation, lower part data of recordings afterwards

The oculomotor experiment consisted of several blocks of trials as shown exemplarily for one subject in Fig 1. Saccade adaptation was induced by using a McLaughlin double step saccade adaptation paradigm [4].

#### Adaptation blocks

A fixation point was presented in the centre of the screen for a duration of 1200 ±200 ms. Subsequently the target reappeared at 10° horizontal position for 80 ms. As soon as the eye started to move the target reappeared at the same position (non-adapted side) or was moved centripetally by an intra-saccadic step of 4° (inward adaptation). Online saccade detection was performed by three-fold criteria: 1) eye velocity of the current sample (N) must have increased by more than 30 deg/s since the previous sample (N-1) and 2) by more than 70 deg/s since the second last sample (N-2). 3) The eye position difference between sample N and N-3 had to be larger than1 deg [36].

The lateral fixation point was presented for 1400–2200 ms and then smoothly moved back to the centre in a sinusoidal manner with a peak velocity of 15°/s in a time period of 500 ms. Smooth retrace of the target was used in order to exclude interference of centripetal saccade to centrifugal saccades. During adaptation blocks only one side was adapted which was randomly defined for each subject before the experiment started. Adaptation blocks consisted of 100 trials with 75 trials in the adapted direction and 25 trials into the non-adapted direction without intra-saccadic displacement. The order of the stimuli was pseudo-randomly intermixed (balancing trials) [37].

#### Baseline blocks

40 trials with 10° target steps were performed, starting with central fixation for 1200 ms ±200 and subsequent peripheral stimulus presentation again for 1200 ms ±200.

#### Extinction blocks

50 trials (25 to the left and 25 to the right) were presented in the same way as described in baseline blocks.

### Data analysis

Horizontal and vertical eye positions were analyzed offline using Matlab (R2012a, The Mathworks Inc., Natick, MA, USA). Eye position data were calibrated and filtered (Gaussian filter, 100 Hz). Eye velocity was calculated as the difference of median eye position of four data points before and after the actual data point.

Eye movements with an angular velocity >30°/s and an amplitude >0.3° were marked as saccades [33] and checked interactively. Saccade amplitude was computed as the difference between eye positions at the end and at the start of a saccade. Saccade latency was computed as the time between the onset of the target shift and the onset of the saccade. Trials were excluded from further analyses because of blinks, anticipatory saccades (latency <70 ms) or extremely long reaction times (latency >800 ms) (3.0% ± 0.7 of the trials of the study in the sleep condition, 6.6% ± 2.4 in the visually deprived condition and 3.8% ±0.9 in the viewing condition).

Saccadic gain was calculated as the ratio between saccade amplitude and amplitude of target displacement. In order to estimate the performance in baseline and extinction blocks a linear regression was computed (by *robustfit* function within Matlab). For the learning process of adaptation exponential fits of the saccade gain were performed using *fminsearch* function within Matlab which uses a Nelder-Mead downhill-simplex algorithm [38]. From the exponential fit saccadic gain was derived after trial #1, 3, 5, 10, 15, 20 and 25 into the direction of adaptation. The regression curve at the beginning and the end of the block are referred to as ‘initial offset’ and ‘final adaptation gain’ hereafter.

In order to quantify individual learning performance we calculated indices for memory retention of motor learning relative to baseline [22]: Percentual gain changes were calculated for these different gains in adaptation and extinction blocks by the formula

$${PGC}_{n}\;=\;\frac{{gain}_{n}-{gain}_{baseline}}{{gain}_{baseline}}*100$$

where n is the selected trial number (see above). The amount of retention [18] after the night of sleep or the 2 hours’ break respectively was calculated by the formula

$$Retention(n)\;=\;\frac{{PGC}_{\left(n\right)\;at\;postadaptation(m)}}{{PGC}_{\left(end\right)\;at\;adaptation2}}*100$$

where *n* indicates the trial number (1–99), *m* the sequence of postadaptations (1 or 2) and *PGC(end) at adaptation 2* refers to the last trial before the (two hours or one night) break (Table 1). Statistical significance was assessed by using analyses of variance (ANOVAs) with the between-subject factor CONDITION (sleep, visual deprivation, viewing condition), the repeated-measures factor TIME for the different time-points of measures of PGC or Retention (at trial #1, 3, 5, 10, 15, 20, 25, 100) and BLOCK for blocks of trials (baseline, adaptation 1, extinction, adaptation 2, post-adaptation 1, post-extinction, post-adaptation 2). The factor SESSION depicts retests after four weeks of subjects that participated in daytime experiments (visual deprivation or viewing condition). Pairwise comparisons were performed by using post-hoc Scheffé tests and T-tests. All tests were performed using SPSS (version 21, IBM Inc. NY/USA). If not stated otherwise, subsequently reported values are means (±1 SE).

## Results

Eye amplitude adapts to final target position in adaptation trials in an exponential manner. Fig 1 shows saccadic gains of a single subject in the experimental condition with visual deprivation. In all analyses the subjects adapted to the new target location; ANOVAs always showed a significant factor *TIME* with p always <0.001. The factor *TIME* is not of particular interest, therefore we will subsequently only report *TIME* x *CONDITION* interactions or differences between conditions.

Sleep in the laboratory was normal (see Table 2) with sleep durations between 427 and 548 minutes (mean ±standard deviation, 494 min ±35). The time needed to sleep onset varied between 6.5 and 36.5 minutes (mean ± standard deviation, 16.4 min ±9.8). Comparisons of average sleep stages in 1st (habituation night) and 2nd night (experimental night) revealed no significant differences (t-Test, p always ≥0.176). The percentage of the sleep stages did not correlate with PGC or retention (p always >0.64).

**Table 2:** Sleep results.

	1^st^ night (habituation)	2^nd^ night (experimental night)
Duration of sleep [min]	483.21 (49.54)	493.80 (35.36)
Sleep onset [min]	15.82 (10.04)	16.37 (9.75)
REM latency [min]	96.54 (56.97)	104.27 (36.46)
SWS latency [min]	22.43 (15.64)	15.23 (4.43)
Awake [%]	0.17 (0.35)	1.23 (2.57)
S1 [%]	8.21 (5.48)	8.04 (10.74)
S2 [%]	50.61 (4.57)	46.67 (10.54)
S3 [%]	9.98 (2.87)	9.42 (2.70)
S4 [%]	10.77 (3.87)	11.39 (4.32)
REM [%]	18.51 (6.35)	21.67 (3.67)
Movement time [%]	1.84 (0.84)	1.76 (0.97)

### Saccadic adaptation before intervention

**Fig 2:**
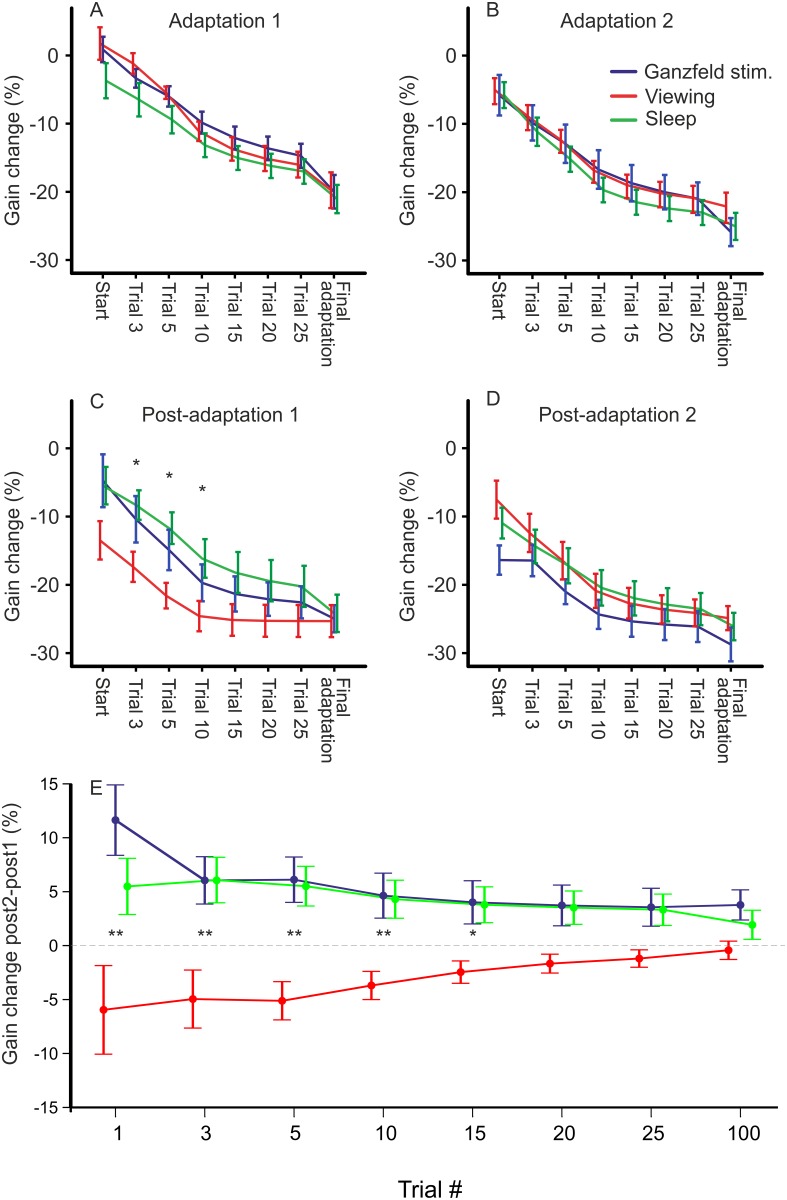
Percentual gain change of all adaptation blocks in relation to baseline gain. In adaptation 1 (A) and 2 (B) subjects show a similar improvement of learning performance in all conditions. After the 2h break/one night of sleep subjects in the free viewing condition show a significantly higher gain reduction than those in the VD condition (C, trial 3 to 10) which diminishes after a short extinction (D). Percentual gain change (E) plotted as the difference between Post-adaptation 2 minus Post-adaptation 1. In the VD conditions adaptation gain was further improved in the second part of the experiment while in the free viewing condition subjects showed a significant loss. Stars indicates significant effects for CONDITION in univariate ANOVAs for each trial depicted (‘*’ = p <0.05, ‘**’ = p <0.01).

Initial saccadic gain during the baseline did not differ between conditions (0.95 (±0.02) in the sleep condition, 0.95 (±0.01) in the viewing condition, 0.95 (±0.02) in the visual deprivation condition; F(2, 32) = 0.022, p = 0.978). Learning progress was the same for all three conditions: there was an increase in learning speed—measured by the percentage of gain change (PGC; see Fig 2)—between adaptation1 and adaptation2: An ANOVA using the factors *TIME*, *CONDITION* and *BLOCK* (adaptation 1 and 2) revealed a main effect for BLOCK (F(1, 32) = 45.4, p <0.001), but no main effect for *CONDITION* (p>0.47) nor an interaction between *BLOCK* x *CONDITION* (p >0.79) or *TIME* x *CONDITION* x *BLOCK* (p >0.39, Fig 2A and 2B). From adaptation1 to adaptation2 subjects improved final adaptation gain in all conditions in a similar manner (ANOVA *BLOCK* x *CONDITION*: main effect BLOCK (F(1, 32) = 39.2, p <0.001; interaction *BLOCK* x *CONDITION* p = 0.12). Separate ANOVAs for each block revealed no significant main effects for CONDITION (adaptation 1: p = 0.35; adaptation 2: p = 0.68) or interactions TIME x CONDITION (adaptation1: p >0.91, adaptation2: p = 0.48). Hence subjects showed highly comparable saccade learning in all conditions.

### First saccade after intervention

**Fig 3:**
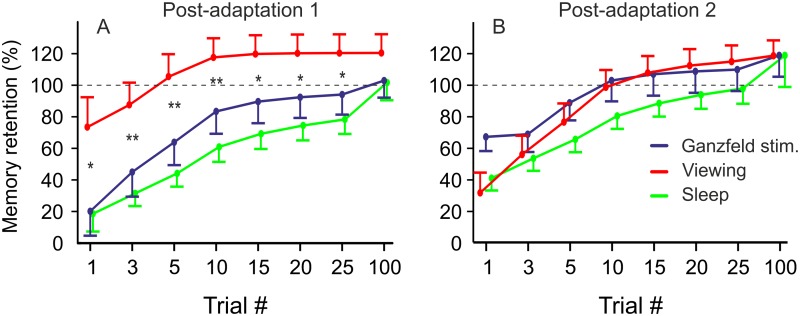
Memory retention of the asymptotic adapted gain at the end of Adaptation 2 achieved in Post-adaptation 1 (A) and 2 (B). In the free viewing condition adapted gain was significantly better memorized compared to the sleep condition and a trend for GS condition. Stars indicate significant effects for CONDITION in univariate ANOVAs for each trial depicted (‘*’ = p <0.05, ‘**’ = p <0.01).

After the intervention (factor CONDITION, i.e. free viewing, visual deprivation, sleep) PGC of the first saccade, i.e. comparison to baseline, failed to reach level of significance (F(2, 31) = 2.03, p = 0.148, Fig 2C). Retention value of the first saccade after intervention, i.e. comparison to adaptation right before intervention, showed significant effect of CONDITION (F(2, 31) = 4.1, p = 0.027, Fig 3A). Post-hoc multiple comparisons revealed a trend for differences between GS and free viewing (mean retention: 20.3% ±15.5, 73.5% ±18.9 respectively; p = 0.075) and significant differences between free viewing and sleep (mean retention sleep condition: 18.6% ±11.3; p = 0.041). GS and sleep condition did not differ (p = 0.997). Note that in the free viewing condition retention was more than three times higher as compared to GS and sleep condition.

### Re-learning after intervention: comparison with previously adapted gain

During post-adaptation subjects rapidly readapted their saccade gain. The retention of previously learned motor adaptation after intervention did not show any *TIME* x *CONDITION* interactions, but *CONDITIONs* differed significantly (F(2, 31) = 5.28, p = 0.011). In post-adaptation 1 subjects in the free viewing condition showed significantly more memory retention compared to those in the GS condition (p = 0.044) and the sleep condition (p = 0.003, Fig 3A). Post-hoc ANOVAs for each level of TIME revealed significant differences between conditions for up to 25 trials. Multiple comparisons showed that free viewing and sleep condition differed significantly (p always <0.039); a trend could be seen for comparisons of subjects in the free viewing condition and the GS condition for trials 3 and 5 (p <0.085). This means that the better memory retention of subjects in the viewing condition right after the intervention remains significant during the first 3 to 25 trials of the block post-adaptation 1.

After post-extinction learning curves of the three conditions were quite similar; analysing PGC, an ANOVA of TIME x CONDITION showed no main effect of CONDITION (p = 0.445) and no significant interaction (p = 0.413). Comparing PGC of post-adaptation 1 and 2, ANOVA revealed a significant effect of *BLOCK* (post-adaptation 1 vs. post-adaptation 2, F(1, 31) = 7.5, p = 0.01) and a significant interaction of *BLOCK* x *CONDITION* (F(2, 31) = 10.4, p <0.001, Fig 2C and 2D). Subtracting PGC post-adaptation 2 minus post-adaptation 1 reveals a memory loss of subjects in the viewing condition compared to the GS and sleep condition: an ANOVA of TIME and CONDITION revealed a significant effect for CONDITION (F(2, 31) = 10.4, p <0.001). Post-hoc ANOVAS for each level of TIME revealed a significant effect for CONDITION for trial 1 to 15 (F(2, 31) always >3.9, p always <0.031). These comparisons showed that subjects in the viewing condition needed 15 trials to regain similar adaptation performance as compared to the other conditions (p always <0.024, Fig 2E).

At the end of post-adaptation 1 and 2 subjects in all conditions reached about the same level of adaptation, hence CONDITION did not differ (post-adaptation 1: F(2, 31) = 0.03, p = 0.97; post-adaptation 2: F(2, 31) = 0.66, p = 0.52).

### Long-term effects

**Fig 4:**
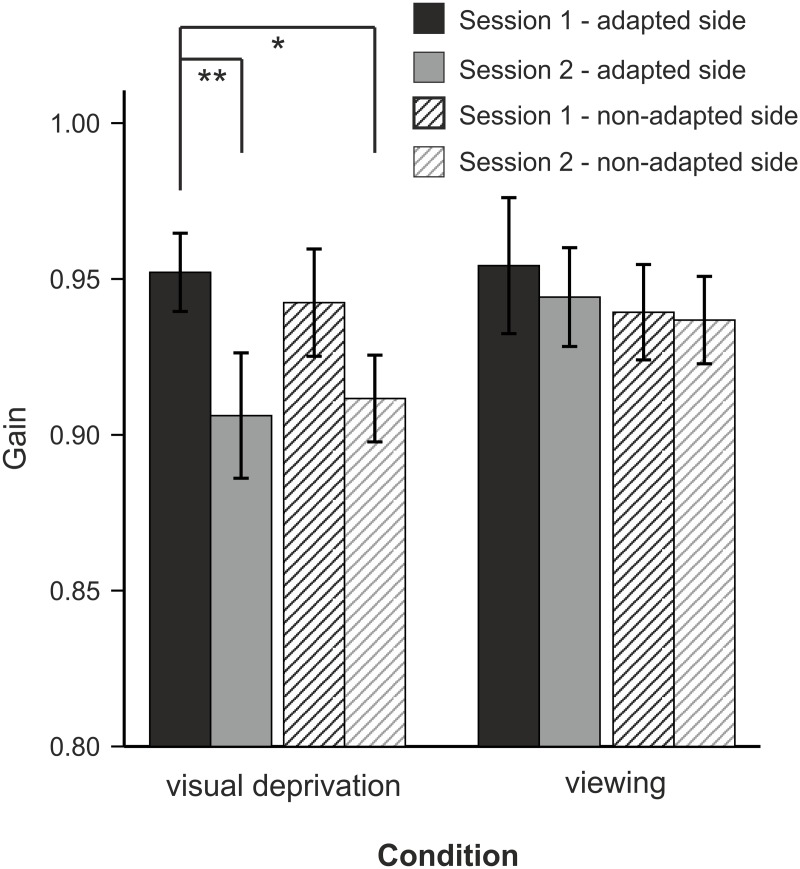
Baseline gain of session 1 (first recording) and session 2 (four weeks later) (mean ±SE). Data are shown for each side separately, i.e. hatched bars belong to the non-adapted side, plain bars to data of the adapted side. Stars indicate significant differences revealed by Student’s T-Test (‘*’ = p <0.05, ‘**’ = p <0.01).

Four weeks after the first measurement participants of the daytime experiment were recorded once again. The baseline gain of the second session was significantly reduced as compared to the first session four weeks ago (ANOVA *SESSION* x *CONDITION* (VD vs. viewing) x *DIRECTION* (adapted vs. non-adapted side)) with a main effect of *SESSION* (F(1, 18) = 9.59, p = 0.006 and an interaction of *SESSION* x *CONDITION* (F(1, 18) = 6.45, p = 0.021, Fig 4). Baseline performance on the adapted side of session 1 of subjects in the VD condition (gain: 0.95 ±0.01) was significantly higher compared with the same side in session 2 (gain: 0.91 ±0.02; T(10) = 3.80, p = 0.003) and with the opposite side (gain: 0.91 ±0.01; T(10) = 3.04, p = 0.013, Fig 4). Gain reduction of the non-adapted side after four weeks did not reach level of significance comparing both sides in session 2 (same side: p = 0.063; opposite side: p = 0.161) with baseline gain in session 1. For the viewing condition there were no significant gain changes (p always >0.47).

Analyses of gain changes during saccade adaptation in session 2 revealed about the same results as in session 1 but parameters of interest did not reach level of significance (p always >0.1).

## Discussion

The aim of this study was to investigate the influence of natural viewing conditions compared to different forms of visual deprivation (VD) on motor memory consolidation. We hypothesized that free viewing following an implicit visuo-motor learning process, i.e. saccadic adaptation, may affect a person’s capacity of retrieving the learned motor behaviour. Therefore, we compared saccadic performance in a paradigm with serial trials of an adaptation—extinction—adaptation sequence before and after two hours of normal viewing and VD, which was either presented by Ganzfeld stimulation (GS) or by a whole night of sleep. Consolidation of learned behaviour, i.e. adaptation retrieval, was assessed by the relearning performance (re-adaptation). The adapted saccadic gain was better preserved following free visual scanning in a natural environment indicating better short term motor memory than after VD. However, this motor memory turned out to be short lasting since the adapted saccadic gain could less be maintained for subjects in the viewing condition: after an additional extinction block saccadic adaptation performance decreased for subjects in the viewing condition whereas in both conditions of VD adaptation performance improved (savings). Hence, VD might contribute to a long term consolidation of the previously adapted saccade gain which was confirmed by the four week follow-up data.

### Impact of rest in normal viewing conditions on adapted saccadic gain

A novel and striking result of our study is an increased storage of adapted gain after two hours of free viewing compared to visual deprivation. Saccadic activities during rest periods show differential effects on motor memory consolidation. Previous studies showed that visual input did not completely extinct adapted motor behaviour even on longer time scales [18,23,39]. Extinction of adapted gain depended on the similarity of the learned adapted saccadic type and vector and the saccade type performed during the rest period [5,13]. In our study subjects in all conditions were exposed to visual stimulation during rest which was completely different from the adaptation condition. In previous studies adapted saccade gain decreased stronger when reflexive saccades to targets without intrasaccadic displacement were performed during rest periods [5,6]. Adapted gain was better maintained when self-induced or even no saccades were performed during rest. In a monkey study Seeberger et al. [6] compared the effects of reflexive saccades during rest with or without visible target after saccade onset and 20 minutes in darkness. Reflexive saccades with visible targets strongly reduced adapted gain whereas invisible targets trials nearly did not reduce adapted gain. These results seem to be in contrast to our study, but there are several differences that may account for it: Reflexive saccades grossly differ from voluntary saccades with or without visual feedback [30]. That means (1) the conditions “reflexive saccade trials with permanent visual targets” (Seeberger et al.) and “free viewing”, i.e. voluntary saccades with potential visual targets in the present study, are of different type. Additionally voluntary saccades without visual targets (GS) differ from reflexive saccades without visual feedback as used by Seeberger et al. [6]; (2) the duration of rest was much longer in our study (2 hours, LTSA) as compared to the monkey study (20 min), i.e. STSA; and (3) the speed of saccade adaptation in monkeys differs from humans.

For the rest period Gaveau et al. [5] have demonstrated that only reflexive saccades of the same amplitude and direction as in the adaptation blocks have been effective to reduce adapted gain, but not self-generated saccades. In our free viewing condition, subjects performed mostly self-generated voluntary saccades, though visually-guided, but not primarily reflexive saccades. Further, voluntary saccades in free viewing are usually smaller than 5–7°, i.e. smaller than the target amplitude used in our adaptation paradigm [40,41]. Thus our finding of a better storage of adapted saccade gain after free viewing (as compared to GS or sleep condition) can be attributed to the fact that saccades performed during free viewing were of totally different type and amplitude than those performed during the adaptation trials. Obviously, the spatial programming of adapted reflexive saccades was not influenced by the programming of voluntary saccades during free viewing. Consistent with this view a study on the execution of consecutive reflexive and voluntary saccades revealed that both types of saccades can be programmed in parallel, probably using a common motor map [42]. Also functional imaging studies showed different cortical substrates activated during the execution of reflexive and voluntary saccades: during voluntary saccades blood oxygen level dependent (BOLD) response was greater in the lateral portions of the frontal eye fields (FEF), in the SEF and the left intraparietal sulcus [43,44]. On the other hand reflexive saccades showed a greater BOLD response in the angular gyrus of the inferior parietal lobe, mostly in the right hemisphere.

Contextual cues during rest may influence savings of saccadic adaptation, e.g. eye position [37,45], head position [46], visual attributes of the target (flickering of the target [47]), target distance [48] or darkness [23]. Hence, saccadic gain can be reproduced and modified best in the experimental condition of learning, i.e. improved but also un-learned in a setting without changing these kind of features. In our study subjects in all conditions were exposed to visual stimulation during rest which was completely different from the adaptation (learning) condition.

### Visual conditions during rest

To date, visual deprivation in saccade adaptation in humans has never been induced by GS or sleep. In non-human primate studies monkeys’ were sitting in a dark room where they were able to sleep [6] or their heads were covered [21]. In human studies participants were asked to close their eyes to achieve visual deprivation [5]. Visual deprivation by eye closure comprises several components that discriminate this condition from free viewing: Apart from absent light stimuli eye movements are not accompanied by visual feedback implying that a visuo-spatial reference frame is missing. This may influence visuo-motor processing in different ways. Brain activity, e.g. differs between open and closed eyes in darkness [49]. Moreover saccadic dynamics differ with the eyes open and closed which has been related to saccade generation during eye closure [30,50]. Accordingly, eye closure seems to affect saccade processing. Changes in luminance intensity also seem to affect saccade-related neural activation [51]. Therefore we kept luminance intensity in our GS design stable. The use of GS as an instrument for visual deprivation allows selectively examining the role of fixation cues on consolidation of adapted saccadic gain.

During sleep lids are usually closed but rapid eye movements (REM) do occur. The question arises as to whether REMs can be compared to voluntary eye movements without visual information. In sleep studies subjects reported processing visual scenes during sleep which they visually explored [30,31]. In a study on REM sleep disorder patients vividly processed their dreams [52]. Likewise visual cortical regions show activation pattern during REM-sleep that resemble pontine-geniculate-occipital (PGO) waves in animals [53,54]. Saccade kinematics revealed that REMs are similar to voluntary eye movements without visual information whereas spontaneous eye movements in darkness were completely different from all other visual conditions [30]. Thus, both, REM-sleep and GS provide about comparable conditions without visual feedback.

### Visual deprivation enables a stable long term consolidation

The second main finding of our study is a stronger relearning of subjects in the VD condition (Ganzfeld stimulation or sleep): in the relearning phase—after a block of adaptation and extinction (post-adaptation 2)—participants of the free viewing condition showed less adapted gain whereas participants of both visually deprived conditions revealed a faster re-learning of adaptation. This indicates better long-term motor memory consolidation of subjects in the VD condition as compared to the free viewing condition. Four weeks later participants of the VD condition still showed remnants of gain adaptation which, however, was not specific to the adapted side.

Previous studies indicated that visual deprivation may facilitate retrieval of adapted gain of saccades. In monkeys adapted saccade gain was partly maintained after one night sleep in a dark cage [55] or after 20 hours blindfoldedness [20]. As previously cited Seeberger et al. [6] studied saccade adaptation before and after blindfoldedness for 20 min. They reported a better maintenance of adapted saccade gain during blindfoldedness compared to extinction trials over this interval. In a related study relearning of saccade adaptation was faster after 30 min of blindfoldedness in a dark cage as compared to 30 min with extinction trials [21].

In a study with human participants visual deprivation elicited a complete storage of adapted saccade gain [5]. In contrast our data show partial maintenance of adapted gain in the first block after rest—but it was less than final adaptation gain before intervention. Our experimental design differed from this study: First, Gaveau et al. [5] used an adaptation paradigm with a centrifugal intra-saccadic target displacement leading to an increased gain (gain up or outward adaptation), whereas in our study gain was decreased by a centripetal target displacement (gain down or inward adaptation). There is an ongoing debate as to whether up and down gain adaptation are related to different neural substrates [13]. Zimmermann [56] found that gain up adaptation shows involves spatial target remapping, while gain down adaptation preferably engages motor learning. Therefore we assume that behavioural differences might be related to the different adaptation paradigms. Second, retention after 15 minutes of rest can be regarded as STSA whereas two hours belong to long-term consolidation (LTSA). Third, visual deprivation by sitting in the dark is different from GS or sleep. Fourth, we investigated complete learning curves of adaptation instead of the first trials only (e.g. [5]). The slope of learning curves over 150 saccades was determined in only one non-human primate study [21]. In line with previous reports our human participants adapted their saccade gain after about 50 trials, i.e. much shorter than in monkeys [1]. We calculated the memory retrieval for several points on the fitting curve. These results are in line with monkey data from Kojima et al. [21]: we showed significant differences between conditions in adaptation performance only in the beginning of the subsequent adaptation course after a 2 hours rest or sleep.

#### Long term motor consolidation of saccadic adaptation

Following rest, the second adaptation block (post-adaptation 2, Table 2) showed an inverse effect: adaptation gain was better maintained and better relearned compared to post-adaptation 1 in the VD conditions. Furthermore remnants of saccadic adaptation were still found after four weeks.

Adapted gain achieved within one single course of adaptation is referred to as short term saccadic adaptation (STSA) [1]. Adapted gain can still be detected 5 days after learning in humans [18]. This may imply diverse learning processes at multiple timescales, which has been suggested in several models of saccadic adaptation [21,27,57] and other types of motor adaptation [19,28,29]. Our short- and long-term results support motor learning of saccade adaptation at multiple timescales, as shown by the context-dependent (VD vs. free viewing) change of saccadic gain over time and exercise. A stable adapted gain based on consolidation of motor memory should be reflected in a high resistance against extinction. Collins and Wallman [57] have demonstrated that prediction of the final target position has a stronger and more long-lasting influence on saccadic adaptation than the retinal error signal. In our experiment adapted gain was more vulnerable to extinction after free viewing than after visual deprivation or sleep. We suggest that subjects mainly rely on visual cues (i.e. retinal error) in the free viewing condition which could make them prone to changes of the visual stimulus in the extinction block. This indicates the role of a stable visual array for resetting adapted saccade amplitudes. In contrast, in our visual deprivation (GS and sleep), subjects are trained to rely on extra-retinal cues, e.g. efference copy or prediction to remap their internal representations of saccade targets. The smaller saccadic adaptation in the first post-adaptation block may be related to reference frame interference since probably because remapping of efference copy signals interfered with remapping of the target shift. In the second post-adaptation block visual cues and efference copy turned out to be unmatched. Therefore, saccadic adaptation following extinction appeared to be mainly influenced by prediction, which is regarded to be the most stable signal for the consolidation of motor memory with respect to the adapted saccade goal [57].

After four weeks we found remnants of saccade adaptation which is in a line with a previous study [22]. Given the known vector-specificity of saccadic adaptation we expected a uni-directional decrease in reflexive saccades but we found a bi-directional decrease. Obviously side specificity diminishes after a longer period of time and remaining gain modification is not related to the vector of the adaptation. We can exclude a non-specific decrease in saccade gain because subjects in the viewing condition did not show a similar decrease in saccade gain as compared to subjects in the GS or sleep condition.

Neurophysiological correlates have been addressed previously: while prediction is generated presumably in cortical areas such as the prefrontal and anterior cingulate cortex, the pre-supplementary eye field and the hippocampus [58], a large number of studies has shown that more downstream cerebellar structures play a crucial role in saccadic adaptation processes [11,13,14,59] which in turn correspond with the posterior parietal cortices for computing visuo-spatial constancy across saccades [60]. When there is no visual feedback following saccadic adaptation, such as during GS or REM sleep in our experiment, trans-saccadic visuo-spatial constancy cannot be maintained sufficiently by visual cues or efference copy, we assume that the cerebellar processing of adaptation is more and more controlled by these prefrontal areas involved in prediction, thereby supporting consolidation of motor memory. This effect may be masked at first by predominant parietal functions of remappping.

#### Sleep does not boost motor memory consolidation in saccadic adaptation

Many motor skills benefit from sleep as their memory consolidation occurs during sleep [32]. This made us suggest that that retention of adapted saccadic gain might benefit from sleep as well. In two studies monkeys were blindfolded for at least one night and re-adapted the next day [20,55], i.e. identical visual deprivation as compared to our sleep condition. Both monkey studies report a large variation of retention on the next day, ranging from no washout to complete washout with an average in-between. Our data are in line with these results. Unfortunately in both studies there were no direct comparisons with free viewing conditions. This comparison can only be deduced from monkey data [6] in which 15 minutes of visual deprivation was applied, where the monkeys showed signs of light sleep. Right after, there was nearly no washout of adapted gain which stands in contrast to related monkey studies [20,55] and to our data. We assume that this might be due to the short duration of sleep and possibly to different sleep stages. To date there is no study which systematically varied saccadic adaptation washout related to the duration of sleep.

In our data we did not find any differences in savings between visual deprivation during wakefulness (GS) and sleep. This might be related to the fact that sleep related memory consolidation holds for explicit motor tasks [61,62]. In contrast, saccadic adaptation is an implicit form of motor learning [1].

Rapid eye movements during sleep might have had an impact on motor adaptation but this was not the case: REM sleep duration was about two hours which is the same amount of time as the GS stimulation duration. Additionally REM sleep duration did not correlate with PGC. Hobson and Friston [63] assume that there are processes of optimization of internal models during REM-sleep that rely on other feedback mechanisms than visual ones, such as proprioception. Indeed, in our experiment visual error was induced visually and hence not of a permanent systemic origin as it may be caused by muscle damage. It has been demonstrated that proprioception of the eye muscles affects vision [64,65] and probably also saccadic adaptation at longer timescales [66]. It is thus possible that sleep contributes to recalibrate the STSA when visual feedback is not available.

Furthermore, in previous studies we could show that sleep improves reaction time of saccades but not the gain [33,67]. In both studies saccadic gain of all participants was almost perfect right from the beginning, i.e. a ceiling effect might have suppressed sleep related motor learning. Now we can show that sleep does not boost motor memory consolidation in saccadic adaptation—at least compared to two hours of visual deprivation in wakefulness.

#### Adapted gain after two hours of free viewing and visual deprivation: a consequence of non-disturbed spatial remapping?

Saccadic gain directly following the rest period was significantly higher after free viewing as compared to VD. In the absence of visual cues in the Ganzfeld or sleep condition, subjects may move their eyes to internally represented target positions [30]. As there is no visual reference frame, remapping of space across each saccade requires updating of target positions by the use of extra-retinal information, i.e. efference copy or prediction. This procedure is neuronally computed mainly by the parietal eye field in the posterior parietal cortex [10,44], and does probably interfere with the remapping of the adapted eye position during the rest period. This could explain why subjects in the GS or sleep condition needed some 50 adaptation trials in the first block after the rest period to regain previous adaptation, at a time, when obviously the influence of prediction was not yet strong enough. The situation in the free viewing condition is different: There is no interference in terms of remapping, as free viewing provides a stable visual reference frame for maintaining spatial constancy across saccades on the basis of visual cues, not requiring the use of extra-retinal cues such as efference copy. Thus we suggest that spatial remapping interferes with memory retention of saccade adaptation in the first block after VD rest period.

## Conclusions

Our data show different effects of visual deprivation and free viewing on a precisely quantifiable motor learning, i.e. saccade adaptation. Adapted saccade gain is best consolidated in a natural viewing condition—if retrieved directly after intervention. In contrast, GS and sleep seem to support long-term consolidation of saccadic adaptation. To some extent our data are contradictory to a previous study [6] but several differences in the study design may have contributed to. Further research is needed to disentangle the influence of visual information during rest.

From our data we speculate that voluntary saccades during free viewing are performed towards stationary visual targets within a stable visual reference frame. This setting does not interfere with the adaptation-induced remapping of visual reflexive saccades during the adaptation trials. In contrast, visual deprivation by Ganzfeld stimulation or sleep does not seem to rely on visual cues (retinal error) and thereby facilitates long term motor memory. It obviously makes adapted gain less vulnerable against extinction, even after four weeks of normal viewing, most probably by using extra-retinal cues for the position of the adapted saccade goal such as prediction. We suggest that VD may play a beneficial role in consolidation of saccadic adaptation as a motor learning program.
